# 4-Chloro-*N*-(2,4-dimethyl­phen­yl)benzene­sulfonamide

**DOI:** 10.1107/S160053681101960X

**Published:** 2011-05-28

**Authors:** K. Shakuntala, Sabine Foro, B. Thimme Gowda

**Affiliations:** a Department of Chemistry, Mangalore University, Mangalagangotri 574 199, Mangalore, India; b Institute of Materials Science, Darmstadt University of Technology, Petersenstrasse 23, D-64287 Darmstadt, Germany

## Abstract

In the title compound, C_14_H_14_ClNO_2_S, the N—H bond points away from the dimethyl­phenyl ring plane. The mol­ecule is twisted at the S atom, with a C—SO_2_—NH—C torsion angle of −75.5 (2)°. The two aromatic rings are tilted relative to each other by 63.3 (1)°. The Cl atom on the chloro­benzene ring is disordered over two sites with site-occupation factors of 0.59 (3) and 0.41 (3), respectively. The crystal structure features inversion-related dimers linked by inter­molecular N—H⋯O hydrogen bonds.

## Related literature

For hydrogen-bonding modes of sulfonamides, see: Adsmond & Grant (2001 ▶). For our studies of the effect of substituents on the structures of *N*-(ar­yl)-amides, see: Gowda *et al.* (2004 ▶), on *N*-(ar­yl)aryl­sulfonamides, see: Shakuntala *et al.* (2011**a* ▶,*b* ▶,c* ▶) and on *N*-(ar­yl)methane­sulfonamides, see: Gowda *et al.* (2007 ▶).
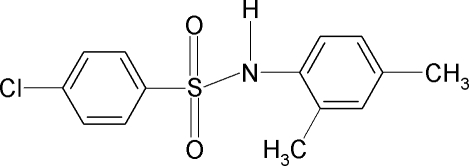


## Experimental

### 

#### Crystal data

C_14_H_14_ClNO_2_S
*M* *_r_* = 295.77
Monoclinic, 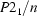

*a* = 8.0493 (7) Å
*b* = 11.4980 (9) Å
*c* = 15.505 (1) Å
β = 90.512 (8)°
*V* = 1434.94 (19) Å^3^
*Z* = 4
Mo *K*α radiation
μ = 0.41 mm^−1^
*T* = 293 K
0.40 × 0.38 × 0.38 mm

#### Data collection

Oxford Diffraction Xcalibur diffractometer with a Sapphire CCD detector
Absorption correction: multi-scan (*CrysAlis RED*; Oxford Diffraction, 2009 ▶) *T*_min_ = 0.854, *T*_max_ = 0.860
5316 measured reflections
2920 independent reflections
2299 reflections with *I* > 2σ(*I*)
*R*_int_ = 0.012

#### Refinement

*R*[*F* ^2^ > 2σ(*F* ^2^)] = 0.037
*wR*(*F* ^2^) = 0.110
*S* = 1.04
2920 reflections
188 parameters
3 restraints
H atoms treated by a mixture of independent and constrained refinement
Δρ_max_ = 0.21 e Å^−3^
Δρ_min_ = −0.28 e Å^−3^

### 

Data collection: *CrysAlis CCD* (Oxford Diffraction, 2009 ▶); cell refinement: *CrysAlis RED* (Oxford Diffraction, 2009 ▶); data reduction: *CrysAlis RED*; program(s) used to solve structure: *SHELXS97* (Sheldrick, 2008 ▶); program(s) used to refine structure: *SHELXL97* (Sheldrick, 2008 ▶); molecular graphics: *PLATON* (Spek, 2009 ▶); software used to prepare material for publication: *SHELXL97*.

## Supplementary Material

Crystal structure: contains datablocks I, global. DOI: 10.1107/S160053681101960X/sj5151sup1.cif

Structure factors: contains datablocks I. DOI: 10.1107/S160053681101960X/sj5151Isup2.hkl

Supplementary material file. DOI: 10.1107/S160053681101960X/sj5151Isup3.cml

Additional supplementary materials:  crystallographic information; 3D view; checkCIF report

## Figures and Tables

**Table 1:** Hydrogen-bond geometry (Å, °)

*D*—H⋯*A*	*D*—H	H⋯*A*	*D*⋯*A*	*D*—H⋯*A*
N1—H1*N*⋯O2^i^	0.83 (2)	2.24 (2)	3.052 (2)	165 (2)

Symmetry code: (i) 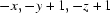.

**Fig. 1.**
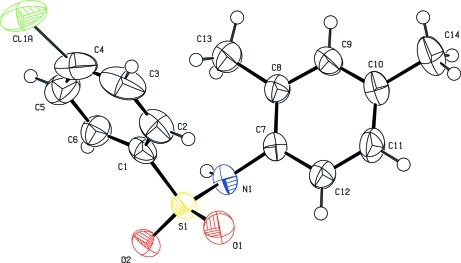
Molecular structure of the title compound, showing the atom labelling scheme. Displacement ellipsoids are drawn at the 50% probability level.

**Fig. 2.**
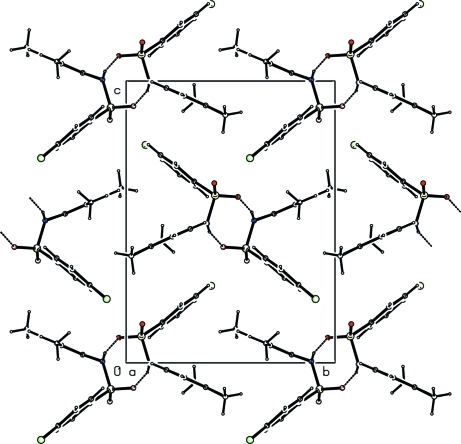
Molecular packing of (I) with hydrogen bonding shown as dashed lines.

## supplementary crystallographic information

### Comment

The hydrogen bonding preferences of sulfonamides have been investigated (Adsmond & Grant, 2001). As part of our work on the substituent effects in the structures of this class of compounds (Gowda *et al.*, 2004, 2007; Shakuntala *et al.*, 2011**a*,*b*,c*), the crystal structure of 4-chloro-*N*-(2,4-dimethylphenyl)-benzenesulfonamide (I) has been determined (Fig.1). In the structure, the amide H atom is *trans* to one of the O atoms of the SO_2_ group. Furthermore, the N—H bond is positioned away from the methyl groups in the aromatic ring.

The molecule is twisted at the S atom with the C—SO_2_—NH—C torsion angle of -75.5 (2)°, compared to the values of -70.3 (3)° in 4-chloro-*N*-(2,3-dimethylphenyl)-benzenesulfonamide (II) (Shakuntala *et al.*, 2011*b*), -70.0 (2)° in 4-chloro-*N*- (2,6-dimethylphenyl)-benzenesulfonamide (III)(Shakuntala *et al.*, 2011*c*), and -53.8 (3)° and -63.4 (3)° in the two independent molecules of 4-chloro-*N*-(phenyl)-benzenesulfonamide (IV) (Shakuntala *et al.*, 2011*a*).

The sulfonyl and the anilino benzene rings are tilted relative to each other by 63.3 (1)° in (I), compared to the values of 34.7 (1)° in (II), 31.9 (1)° in (III), and 69.1 (1)° and 82.6 (1)° in the two independent molecules of (IV).

The packing of molecules into dimers in the title compound *via* intermolecular N—H···O hydrogen bonds (Table 1) is shown in Fig. 2.

### Experimental

A solution of chlorobenzene (10 ml) in chloroform (40 ml) was treated dropwise with chlorosulfonic acid (25 ml) at 0 ° C. After the initial evolution of hydrogen chloride subsided, the reaction mixture was brought to room temperature and poured into crushed ice in a beaker. The chloroform layer was separated, washed with cold water and allowed to evaporate slowly. The residual 4-chlorobenzenesulfonylchloride was treated with 2,4-dimethylaniline in the stoichiometric ratio and boiled for ten minutes. The reaction mixture was then cooled to room temperature and added to ice cold water (100 ml). The resulting 4-chloro-*N*-(2,4-dimethylphenyl)-benzenesulfonamide was filtered under suction and washed thoroughly with cold water. It was then recrystallized to constant melting point from aqueous ethanol The compound was characterized by recording its infrared and NMR spectra.

Prism like colorless single crystals used in X-ray diffraction studies were grown in ethanolic solution by slow evaporation at room temperature.

### Refinement

The H atom of the NH group was located in a difference map and its coordinates were refined with the N—H  distance restrained to 0.86 (2) Å. The other H atoms were positioned with idealized geometry using a riding model with the aromatic C—H = 0.93 Å and methyl C—H = 0.96 Å. All H atoms were refined with isotropic displacement parameters (set to 1.2 times of the *U*_eq_ of the parent atom). Atom CL1 is disordered and was refined using a split model. The corresponding site-occupation factors were refined so that their sum was unity with occupancy factors converging to 0.59 (3) and 0.41 (3). The corresponding bond distances in the disordered group were restrained to be equal.

### Crystal data

**Table:**

C_14_H_14_ClNO_2_S	*F*(000) = 616
*M**_r_* = 295.77	*D*_x_ = 1.369 Mg m^−^^3^
Monoclinic, *P*2_1_/*n*	Mo *K*α radiation, λ = 0.71073 Å
Hall symbol: -P 2yn	Cell parameters from 2311 reflections
*a* = 8.0493 (7) Å	θ = 2.6–27.8°
*b* = 11.4980 (9) Å	µ = 0.41 mm^−^^1^
*c* = 15.505 (1) Å	*T* = 293 K
β = 90.512 (8)°	Prism, colourless
*V* = 1434.94 (19) Å^3^	0.40 × 0.38 × 0.38 mm
*Z* = 4	

### Data collection

**Table:**

Oxford Diffraction Xcalibur diffractometer with a Sapphire CCD detector	2920 independent reflections
Radiation source: fine-focus sealed tube	2299 reflections with *I* > 2σ(*I*)
graphite	*R*_int_ = 0.012
ω scans	θ_max_ = 26.4°, θ_min_ = 2.6°
Absorption correction: multi-scan (*CrysAlis RED*; Oxford Diffraction, 2009)	*h* = −10→9
*T*_min_ = 0.854, *T*_max_ = 0.860	*k* = −10→14
5316 measured reflections	*l* = −19→11

### Refinement

**Table:**

Refinement on *F*^2^	Secondary atom site location: difference Fourier map
Least-squares matrix: full	Hydrogen site location: inferred from neighbouring sites
*R*[*F*^2^ > 2σ(*F*^2^)] = 0.037	H atoms treated by a mixture of independent and constrained refinement
*wR*(*F*^2^) = 0.110	*w* = 1/[σ^2^(*F*_o_^2^) + (0.0596*P*)^2^ + 0.340*P*] where *P* = (*F*_o_^2^ + 2*F*_c_^2^)/3
*S* = 1.04	(Δ/σ)_max_ = 0.001
2920 reflections	Δρ_max_ = 0.21 e Å^−^^3^
188 parameters	Δρ_min_ = −0.28 e Å^−^^3^
3 restraints	Extinction correction: *SHELXL97* (Sheldrick, 2008), Fc^*^=kFc[1+0.001xFc^2^λ^3^/sin(2θ)]^-1/4^
Primary atom site location: structure-invariant direct methods	Extinction coefficient: 0.019 (2)

### Special details

**Table:**

Experimental. CrysAlis RED (Oxford Diffraction, 2009) Empirical absorption correction using spherical harmonics, implemented in SCALE3 ABSPACK scaling algorithm.
Geometry. All e.s.d.'s (except the e.s.d. in the dihedral angle between two l.s. planes) are estimated using the full covariance matrix. The cell e.s.d.'s are taken into account individually in the estimation of e.s.d.'s in distances, angles and torsion angles; correlations between e.s.d.'s in cell parameters are only used when they are defined by crystal symmetry. An approximate (isotropic) treatment of cell e.s.d.'s is used for estimating e.s.d.'s involving l.s. planes.
Refinement. Refinement of *F*^2^ against ALL reflections. The weighted *R*-factor *wR* and goodness of fit *S* are based on *F*^2^, conventional *R*-factors *R* are based on *F*, with *F* set to zero for negative *F*^2^. The threshold expression of *F*^2^ > σ(*F*^2^) is used only for calculating *R*-factors(gt) *etc*. and is not relevant to the choice of reflections for refinement. *R*-factors based on *F*^2^ are statistically about twice as large as those based on *F*, and *R*- factors based on ALL data will be even larger.

### Fractional atomic coordinates and isotropic or equivalent isotropic displacement parameters (Å^2^)

**Table:**

	*x*	*y*	*z*	*U*_iso_*/*U*_eq_	Occ. (<1)
C1	0.0604 (2)	0.32592 (15)	0.64405 (11)	0.0432 (4)	
C2	0.1241 (3)	0.24160 (19)	0.69852 (14)	0.0618 (6)	
H2	0.2380	0.2366	0.7085	0.074*	
C3	0.0167 (4)	0.1645 (2)	0.73809 (15)	0.0796 (7)	
H3	0.0575	0.1067	0.7745	0.096*	
C4	−0.1506 (4)	0.1748 (2)	0.72276 (14)	0.0746 (7)	
C5	−0.2145 (3)	0.2570 (2)	0.66818 (15)	0.0679 (6)	
H5	−0.3284	0.2615	0.6583	0.082*	
C6	−0.1083 (2)	0.33272 (18)	0.62807 (13)	0.0535 (5)	
H6	−0.1499	0.3885	0.5902	0.064*	
C7	0.3261 (2)	0.29199 (15)	0.47078 (11)	0.0388 (4)	
C8	0.2602 (2)	0.18976 (17)	0.43790 (12)	0.0467 (4)	
C9	0.3707 (2)	0.10523 (17)	0.40995 (14)	0.0533 (5)	
H9	0.3280	0.0361	0.3878	0.064*	
C10	0.5409 (2)	0.11919 (19)	0.41361 (13)	0.0515 (5)	
C11	0.6010 (2)	0.2211 (2)	0.44796 (14)	0.0581 (5)	
H11	0.7152	0.2320	0.4527	0.070*	
C12	0.4960 (2)	0.30731 (18)	0.47550 (13)	0.0508 (5)	
H12	0.5395	0.3763	0.4974	0.061*	
C13	0.0765 (2)	0.1693 (2)	0.43064 (19)	0.0756 (7)	
H13A	0.0278	0.2267	0.3932	0.091*	
H13B	0.0276	0.1748	0.4867	0.091*	
H13C	0.0564	0.0932	0.4073	0.091*	
C14	0.6541 (3)	0.0252 (2)	0.37959 (17)	0.0733 (7)	
H14A	0.6704	0.0365	0.3189	0.088*	
H14B	0.6044	−0.0495	0.3891	0.088*	
H14C	0.7593	0.0290	0.4091	0.088*	
N1	0.22067 (18)	0.38712 (14)	0.49490 (10)	0.0422 (4)	
H1N	0.135 (2)	0.3982 (17)	0.4655 (12)	0.051*	
O1	0.34964 (17)	0.42218 (13)	0.63764 (9)	0.0598 (4)	
O2	0.10520 (16)	0.53494 (11)	0.58902 (9)	0.0535 (4)	
Cl1A	−0.3124 (18)	0.0933 (11)	0.7734 (2)	0.086 (2)	0.59 (3)
Cl1B	−0.241 (3)	0.0629 (10)	0.7776 (4)	0.089 (3)	0.41 (3)
S1	0.19347 (5)	0.42678 (4)	0.59428 (3)	0.04223 (16)	

### Atomic displacement parameters (Å^2^)

**Table:**

	*U*^11^	*U*^22^	*U*^33^	*U*^12^	*U*^13^	*U*^23^
C1	0.0585 (11)	0.0365 (9)	0.0347 (9)	0.0036 (8)	0.0073 (8)	−0.0025 (7)
C2	0.0819 (15)	0.0539 (12)	0.0495 (12)	0.0129 (11)	0.0006 (10)	0.0053 (10)
C3	0.1420 (18)	0.0483 (13)	0.0485 (12)	−0.0011 (15)	0.0017 (14)	0.0120 (10)
C4	0.1281 (16)	0.0603 (14)	0.0356 (11)	−0.0405 (15)	0.0183 (12)	−0.0085 (10)
C5	0.0748 (15)	0.0753 (15)	0.0539 (13)	−0.0250 (12)	0.0108 (11)	−0.0042 (12)
C6	0.0553 (11)	0.0544 (12)	0.0508 (11)	−0.0032 (9)	0.0047 (9)	0.0063 (9)
C7	0.0369 (8)	0.0433 (10)	0.0363 (9)	0.0056 (7)	0.0035 (7)	0.0014 (7)
C8	0.0362 (9)	0.0505 (11)	0.0533 (11)	0.0013 (8)	0.0010 (8)	−0.0058 (9)
C9	0.0505 (11)	0.0487 (11)	0.0609 (13)	0.0032 (9)	0.0015 (9)	−0.0143 (9)
C10	0.0457 (10)	0.0594 (12)	0.0495 (11)	0.0153 (9)	0.0033 (8)	−0.0033 (9)
C11	0.0338 (9)	0.0745 (15)	0.0662 (13)	0.0062 (9)	0.0009 (9)	−0.0114 (11)
C12	0.0382 (9)	0.0549 (11)	0.0592 (12)	−0.0031 (8)	0.0032 (8)	−0.0103 (10)
C13	0.0415 (11)	0.0741 (16)	0.111 (2)	−0.0044 (10)	0.0007 (12)	−0.0314 (15)
C14	0.0639 (13)	0.0792 (17)	0.0769 (16)	0.0278 (12)	0.0067 (11)	−0.0135 (13)
N1	0.0404 (8)	0.0463 (8)	0.0400 (8)	0.0099 (7)	0.0027 (6)	0.0002 (7)
O1	0.0516 (8)	0.0712 (10)	0.0565 (9)	0.0007 (7)	−0.0053 (6)	−0.0125 (7)
O2	0.0587 (8)	0.0366 (7)	0.0655 (9)	0.0055 (6)	0.0118 (7)	−0.0034 (6)
Cl1A	0.123 (4)	0.088 (3)	0.0468 (7)	−0.057 (3)	0.0104 (13)	0.0026 (10)
Cl1B	0.135 (7)	0.076 (2)	0.0556 (12)	−0.045 (3)	0.015 (2)	0.0044 (13)
S1	0.0442 (3)	0.0389 (3)	0.0437 (3)	0.00330 (18)	0.00397 (18)	−0.00408 (19)

### Geometric parameters (Å, °)

**Table:**

C1—C6	1.380 (3)	C9—C10	1.380 (3)
C1—C2	1.382 (3)	C9—H9	0.9300
C1—S1	1.7613 (18)	C10—C11	1.374 (3)
C2—C3	1.385 (3)	C10—C14	1.511 (3)
C2—H2	0.9300	C11—C12	1.373 (3)
C3—C4	1.371 (4)	C11—H11	0.9300
C3—H3	0.9300	C12—H12	0.9300
C4—C5	1.366 (4)	C13—H13A	0.9600
C4—Cl1B	1.710 (5)	C13—H13B	0.9600
C4—Cl1A	1.792 (5)	C13—H13C	0.9600
C5—C6	1.373 (3)	C14—H14A	0.9600
C5—H5	0.9300	C14—H14B	0.9600
C6—H6	0.9300	C14—H14C	0.9600
C7—C12	1.380 (2)	N1—S1	1.6236 (16)
C7—C8	1.385 (3)	N1—H1N	0.831 (15)
C7—N1	1.436 (2)	O1—S1	1.4212 (15)
C8—C9	1.389 (3)	O2—S1	1.4343 (13)
C8—C13	1.501 (3)		
			
C6—C1—C2	120.59 (19)	C11—C10—C9	117.48 (17)
C6—C1—S1	119.02 (14)	C11—C10—C14	122.26 (19)
C2—C1—S1	120.39 (16)	C9—C10—C14	120.3 (2)
C1—C2—C3	119.3 (2)	C12—C11—C10	121.37 (17)
C1—C2—H2	120.3	C12—C11—H11	119.3
C3—C2—H2	120.3	C10—C11—H11	119.3
C4—C3—C2	118.9 (2)	C11—C12—C7	120.26 (18)
C4—C3—H3	120.5	C11—C12—H12	119.9
C2—C3—H3	120.5	C7—C12—H12	119.9
C5—C4—C3	122.1 (2)	C8—C13—H13A	109.5
C5—C4—Cl1B	131.9 (10)	C8—C13—H13B	109.5
C3—C4—Cl1B	105.8 (10)	H13A—C13—H13B	109.5
C5—C4—Cl1A	111.2 (6)	C8—C13—H13C	109.5
C3—C4—Cl1A	126.6 (6)	H13A—C13—H13C	109.5
Cl1B—C4—Cl1A	22.0 (4)	H13B—C13—H13C	109.5
C4—C5—C6	119.1 (2)	C10—C14—H14A	109.5
C4—C5—H5	120.4	C10—C14—H14B	109.5
C6—C5—H5	120.4	H14A—C14—H14B	109.5
C5—C6—C1	119.9 (2)	C10—C14—H14C	109.5
C5—C6—H6	120.1	H14A—C14—H14C	109.5
C1—C6—H6	120.1	H14B—C14—H14C	109.5
C12—C7—C8	120.24 (16)	C7—N1—S1	123.10 (12)
C12—C7—N1	118.46 (16)	C7—N1—H1N	117.4 (14)
C8—C7—N1	121.16 (15)	S1—N1—H1N	111.2 (14)
C7—C8—C9	117.70 (16)	O1—S1—O2	119.66 (9)
C7—C8—C13	122.31 (17)	O1—S1—N1	108.20 (8)
C9—C8—C13	119.98 (18)	O2—S1—N1	105.10 (8)
C10—C9—C8	122.93 (19)	O1—S1—C1	107.88 (9)
C10—C9—H9	118.5	O2—S1—C1	107.04 (8)
C8—C9—H9	118.5	N1—S1—C1	108.58 (8)
			
C6—C1—C2—C3	−0.9 (3)	C8—C9—C10—C11	1.1 (3)
S1—C1—C2—C3	178.87 (16)	C8—C9—C10—C14	−178.1 (2)
C1—C2—C3—C4	−0.7 (3)	C9—C10—C11—C12	−1.7 (3)
C2—C3—C4—C5	1.5 (4)	C14—C10—C11—C12	177.5 (2)
C2—C3—C4—Cl1B	177.4 (3)	C10—C11—C12—C7	1.4 (3)
C2—C3—C4—Cl1A	−174.5 (4)	C8—C7—C12—C11	−0.3 (3)
C3—C4—C5—C6	−0.8 (4)	N1—C7—C12—C11	−175.95 (18)
Cl1B—C4—C5—C6	−175.4 (4)	C12—C7—N1—S1	−73.9 (2)
Cl1A—C4—C5—C6	175.7 (3)	C8—C7—N1—S1	110.48 (18)
C4—C5—C6—C1	−0.8 (3)	C7—N1—S1—O1	41.30 (16)
C2—C1—C6—C5	1.6 (3)	C7—N1—S1—O2	170.21 (14)
S1—C1—C6—C5	−178.15 (16)	C7—N1—S1—C1	−75.53 (16)
C12—C7—C8—C9	−0.4 (3)	C6—C1—S1—O1	164.67 (15)
N1—C7—C8—C9	175.19 (17)	C2—C1—S1—O1	−15.08 (18)
C12—C7—C8—C13	−179.4 (2)	C6—C1—S1—O2	34.68 (17)
N1—C7—C8—C13	−3.9 (3)	C2—C1—S1—O2	−145.06 (16)
C7—C8—C9—C10	−0.1 (3)	C6—C1—S1—N1	−78.29 (16)
C13—C8—C9—C10	179.0 (2)	C2—C1—S1—N1	101.96 (16)

### Hydrogen-bond geometry (Å, °)

**Table:**

*D*—H···*A*	*D*—H	H···*A*	*D*···*A*	*D*—H···*A*
N1—H1N···O2^i^	0.83 (2)	2.24 (2)	3.052 (2)	165 (2)

Symmetry codes: (i) −*x*, −*y*+1, −*z*+1.
